# Perspectives for Clinical Translation of Adipose Stromal/Stem Cells

**DOI:** 10.1155/2019/5858247

**Published:** 2019-05-02

**Authors:** Mimmi Patrikoski, Bettina Mannerström, Susanna Miettinen

**Affiliations:** ^1^Adult Stem Cell Group, Faculty of Medicine and Health Technology, Tampere University, 33520 Tampere, Finland; ^2^Research, Development and Innovation Centre, Tampere University Hospital, 33520 Tampere, Finland; ^3^Research Program for Clinical and Molecular Metabolism, Faculty of Medicine, University of Helsinki, 00014, Finland; ^4^Department of Oral and Maxillofacial Diseases, University of Helsinki and Helsinki University Hospital, 00014, Finland

## Abstract

Adipose stromal/stem cells (ASCs) are an ideal cell type for regenerative medicine applications, as they can easily be harvested from adipose tissue in large quantities. ASCs have excellent proliferation, differentiation, and immunoregulatory capacities that have been demonstrated in numerous studies. Great interest and investment have been placed in efforts to exploit the allogeneic use and immunomodulatory and anti-inflammatory effects of ASCs. However, bridging the gap between *in vitro* and *in vivo* studies and moving into clinical practice remain a challenge. For the clinical translation of ASCs, several issues must be considered, including how to characterise such a heterogenic cell population and how to ensure their safety and efficacy. This review explores the different phases of *in vitro* and preclinical ASC characterisation and describes the development of appropriate potency assays. In addition, good manufacturing practice requirements are discussed, and cell-based medicinal products holding marketing authorisation in the European Union are reviewed. Moreover, the current status of clinical trials applying ASCs and the patent landscape in the field of ASC research are presented. Overall, this review highlights the applicability of ASCs for clinical cell therapies and discusses their potential.

## 1. Introduction

Cell-based therapies are a novel approach to treat medical conditions that have limited or no effective therapeutic options. Adipose tissue-derived multipotent cells known as adipose stromal/stem cells (ASCs) are particularly promising candidates for diverse clinical applications, owing to their excellent proliferation and differentiation capacity [1, 2], low immunogenicity [3, 4], and ability for immunomodulation [3, 5–9]. Great interest has been directed to allogeneic use, immunomodulatory therapies, and therapies taking advantage of the paracrine effects of ASCs. ASCs are already being used in clinical applications, e.g., for the treatment of autoimmune diseases such as Crohn's disease [10], and as a regenerative therapy for craniomaxillofacial bone defects [11].

Stem cells are defined by two basic properties: the ability to self-renew and the ability to differentiate into one or more specialised cell types [12, 13]. Stromal cells are multipotent progenitor cells that are found in the connective tissue of any organ with the limited ability to proliferate and differentiate into one or several specific cell types [14]. Careful characterisation of an ASC population is the first step towards determining its viability for clinical applications. Heterogeneity is a characteristic of ASCs, necessitating their careful *in vitro* and preclinical characterisation. If cells are expanded prior to clinical use, the appropriate cell expansion protocol(s) must be determined, as they can have an effect on ASC characteristics [15]. Foetal bovine serum (FBS) has traditionally been utilised for ASC culture [2, 16–18], despite the safety concerns associated with its clinical use. Alternatives to FBS use include autologous or allogeneic human serum (HS) [19–21] or platelet lysate- (PL-) based cultures [22, 23], as well as completely xeno-free/serum-free (XF/SF) cultures [21, 24–27]. The search for optimal conditions for *in vitro* cell expansion remains ongoing, which is evident from the variation of culture conditions used in current clinical trials.

The use of autologous versus allogeneic ASCs is a relevant question when developing clinical therapies. Currently, many clinical studies are carried out using autologous ASCs, which causes high variation in clinical outcome. Several factors affect ASC characteristics, including donor age, gender and weight, and the anatomic harvest location and depth [28, 29]. Therefore, the use of allogenic cells would be more straightforward from a practical point of view, since they could be isolated and fully characterised prior to clinical use.

Potency assays are useful tools for the characterisation of ASCs for clinical use. We present some of the analytic methods that can be utilised as potency assays. The development of appropriate mechanism of action- (MOA-) based potency assays is important for confirming a cell product's efficacy. This is often required by regulatory authorities but is also essential as a quality control method for ensuring reproducibility of the production protocol. Moreover, preclinical *in vivo* studies are mandatory for progressing to clinical trials, although investigating human cells in animal models is always challenging. Further, we discuss safety aspects related to clinical translations, such as genomic stability of ASCs and the effects of paracrine signals in facilitating the formation of a tumour microenvironment.

In this review, we also shed light on the current status of clinical trials, investigating ASCs, included in the context of the patenting landscape in the field of ASC research. A total of 244 clinical trials were registered on the http://www.clinicaltrials.gov database in September 2018 to evaluate the potential of ASCs for treating various diseases. The number of clinical trials on ASCs has been steadily increasing during the past decade, from nine registered trials in 2009 to 244 in 2018 (25-fold increase). However, most registered clinical trials are still in phase I, and only five cell-based medicinal products currently hold marketing authorisation in the European Union (EU). Moreover, an increased number of patents have been filed by universities as they become more involved in clinical trials and commercialisation. We conducted a patent search with keywords “adipose stem cell” in the Espacenet Worldwide database (http://www.epo.org) and found 863 hits. ASCs hold a great potential for treating several diseases, and thus, capacity for translation from the research phase to routine cell-based treatments should be strengthened. The present review encourages this translation and defines critical aspects when aiming at the clinical use of ASCs.

## 2. Adipose Stromal/Stem Cells

**Figure 1:**
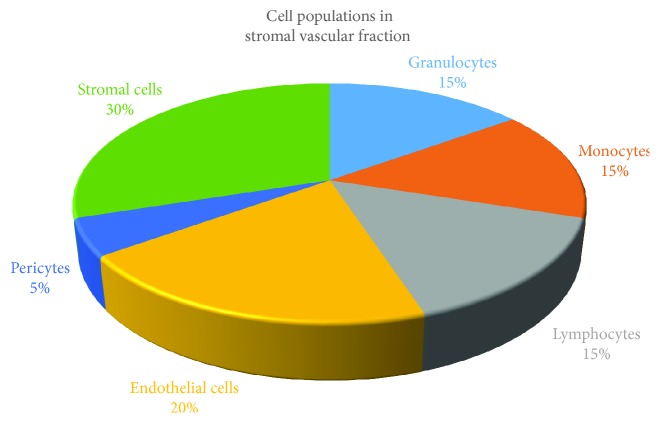
Cell populations in stromal vascular fraction (SVF) [14]. The SVF contains a heterogeneous mesenchymal cell population, e.g., cells of endothelial, hematopoietic, and pericytic origin, among others. Cells of hematopoietic origin include granulocytes (15%), monocytes (15%), lymphocytes (15%), and stem and progenitor cells (<0.1%). Additionally, endothelial cells (20%), pericytes (50%), and stromal cells (30%) are found in SVF.

Human ASCs are multipotent progenitor cells found in adult adipose tissue [2]. After digestion by collagenase, adipose tissue is divided into an adipocyte fraction and a stromal vascular fraction (SVF) (Figure 1), from which ASCs are selected based on their plastic adherence property. For over 15 years, adipose tissue has been extensively studied as a cell source for tissue engineering and regenerative medicine [2, 17, 30–32]. ASCs are primarily mesodermal, but some are of neural crest (ectodermal) origin [33], and have the potential to differentiate into at least adipogenic, chondrogenic, and osteogenic cells [1, 2]. Additionally, ASCs have the ability to reduce inflammation, mediated primarily via paracrine effects [6, 7, 9, 34, 35]. Both SVF and ASCs are currently utilised in clinics, but selecting between these two should be based on the particular disease application. The SVF is especially used for soft tissue reconstruction [36], whereas expanded ASCs might be selected for applications where a larger cell dose is required [37]. Additionally, SVF is reported to be more heterogeneous compared with culture-expanded ASCs that are more homogeneous [38].

A critical discussion has been ongoing lately within the scientific community on the origin, developmental potential, and biological functions of these cells [33, 39]. The terms “mesenchymal stem cell (MSC)” and “adipose stem cell (ASC)” are under scrutiny over how accurately they describe the origin and stemness of the cells. It has therefore been suggested that the terms “tissue-specific progenitor cells” or “medicinal signalling cells” may be more appropriate for these heterogeneous groups of fibroblastic-like stromal cells [33, 39]. The discussion is welcome and relevant in order to clarify the terminology and to avoid overstatements on the cells' potential. Nevertheless, the terms ASCs and MSCs are still widely used in the scientific community, and also in this review, we use the term ASC for adipose tissue-derived stromal/stem cells and the term MSC for all types of mesenchymal stromal/stem cells derived from bone marrow, adipose tissue, and other tissue sources.

### 2.1. ASC Characteristics Are Dependent on Cell Donor and Tissue Source

Cell characteristics vary significantly between donors, which makes selection of the donor an important concern and justifies the use of allogeneic ASCs in clinical applications. For example, the proliferation and differentiation capacity of ASCs may be affected by various factors, such as the age, sex, or body mass index of the adipose tissue donor [40–42]. For example, a negative correlation between high donor age and proliferation and differentiation efficiency has been observed [43], and high body mass index also seems to reduce the proliferation capacity and compromise the osteogenic potential of ASCs [42].

Adipose tissue is widely dispersed in humans. It has been suggested that each fat depot has distinct developmental origins [33, 39], which will potentially affect cell characteristics that are critical in the context of expansion, differentiation, and therapeutics [44]. For instance, it has been shown that ASCs derived from distinct visceral fat depots are remarkably heterogeneous, and gene expression profiles and differentiation capabilities differ significantly between ASCs derived from different fat depots [44]. Furthermore, the depth of adipose tissue harvest appears to be critical on ASC proliferation and adipogenic potential, as ASCs from subcutaneous adipose tissue have increased proliferation and adipogenic capacities compared to ASCs of visceral origin [28].

Moreover, ASCs from males seem to have greater osteogenic capacity [29]. Interestingly, ASCs derived from obese and diabetic subjects have shown reduced capacity for immunomodulation, suggesting that the local microenvironment of donor tissue impacts their anti-inflammatory functions [45]. ASCs derived from obese and diabetic subjects have been shown to exhibit a reduction in typical immunosuppressive activities and be less effective in suppressing lymphocyte proliferation that activates the M2 macrophage phenotype than lean-derived ASCs [45].

In addition to biological factors, the isolation method used, such as abdominoplasty, liposuctions, or specific devices, may have an effect on a selected cell population [38]. It has been reported that liposuction provides fewer ASCs compared to excised fat tissue but that cell proliferation is higher from liposuction, and significantly more cells display MSC markers [46, 47]. Nevertheless, ASCs obtained using both isolation methods have equivalent viability and differentiation capacity.

## 3. Culture of ASCs

When ASCs are used in clinical applications, cell expansion is often needed to obtain a clinically relevant cell number, in the range of millions to hundreds of millions. The required cell number is estimated via dose escalation studies and is always dependent on the disease application and especially the defect size. Moreover, cell dose may depend on the administration method, i.e., whether cells are locally carried to the defect site on a scaffold or infused into the bloodstream, in which case the cell dose is often estimated in relation to patient weight.

According to good manufacturing practices (GMP), ex vivo cell expansion should be reproducible, robust, and efficient. To meet these criteria, fully defined culture conditions would be needed in order to enable efficient cell proliferation and maintenance of basic stem cell characteristics [48]. This section introduces traditional serum-based culture conditions (FBS versus HS) for ASCs and discusses the alternative options of XF and/or SF cultures, such as PL-based cultures or fully defined XF/SF culture conditions.

### 3.1. Standard ASC Cultures

*In vitro* culture of ASCs requires optimal conditions that support both proliferation and differentiation when induced. Traditionally, cell culture media consisted of a basal medium, such as alpha-modified Eagle's medium (*α*-MEM) or Dulbecco's modified Eagle's medium/Ham's F12 (DMEM/F-12) supplemented with 10% serum, 1% antibiotics (usually penicillin and/or streptomycin), and 1% L-glutamine [2, 49]. Moreover, FBS has routinely been used in ASC cultures [2, 16, 17] because it provides a cocktail of growth factors, cytokines, adhesion proteins, and other nutrients to the cells [50].

### 3.2. Xeno-Free Cultures

For clinical cell therapies, all animal-derived components should be replaced with XF alternatives. If ASCs are cultured in the presence of FBS, there is a potential for zoonoses to be transferred to the patient, which could cause severe sequelae related to xenogeneic infections [51, 52]. In successive administration of cells, antibodies towards bovine antigens may also be produced, which can affect the efficacy of cell-based treatments. Due to these concerns, there is growing interest in developing novel cultivation media for ASCs, although validated batches of FBS have been accepted for ongoing clinical trials by the regulatory authorities. However, various XF and/or SF alternatives have been developed and studied for ASC cultures, but relatively few formulations are commercially available for clinical use.

Allogeneic HS is an XF alternative for FBS with similar properties. Our group and others have shown that supplementation with HS enhances or promotes an equivalent effect on ASC doubling time compared to FBS [19–21, 53], with no substantial differences in cell morphology or immunophenotype observed between HS and FBS conditions [19, 54]. Moreover, greater proliferation rates and more efficient osteogenic differentiation capacity have been demonstrated in HS medium compared to FBS medium [19, 21, 53, 55]. However, batch-to-batch variation between serum-supplemented media affects the proliferation rate and differentiation capacity of the cells [56]. Therefore, the safety and quality of transplanted ASCs can be enhanced by replacing undefined and/or animal-derived components with fully defined GMP-compliant XF/SF reagents [15, 21, 24, 57]. Validated batches of autologous and allogeneic HS have been used also in clinical studies [11].

One approach to replace FBS from cell culture is to use human platelet-derived supplements [23]. Schallmoser and Strunk [58] introduced a standard protocol for the preparation of pooled human PL. A reservoir of growth factors and cytokines stored in platelet granules can be released by freeze/thaw cycles, sonication, or chemical treatment [23]. The physiological role of platelets in wound healing and tissue repair is a basis for using human platelet derivatives in regenerative medicine. Several studies on ASC culture in PL-based medium have been published. In most of these studies, ASCs exhibited high proliferation rates, maintained multipotency and differentiation capacity, and showed stable chromosomes when cultured in PL-based medium, supporting the use of PL for cell expansion in clinical studies [22, 23, 59–63]. Although some contradictory results showing decreased population doubling times in PL cultures exist [64], the majority of reports support the use of PL in ASC culture. PL-based cultures are also currently being used in clinical trials [65].

### 3.3. Serum-Free Cultures

Completely serum component-free culture conditions for ASCs have been investigated, but only a few studies exist in which cell isolation, expansion, and differentiation were performed using only XF/SF reagents. Our group was the first to publish a successful and comprehensive set of XF/SF isolation, expansion, and multilineage differentiation protocols for ASCs [15]. Moreover, encouraging results on ASC culture in chemically defined SF media have been published by several other researchers [21, 24–26, 66]. In the majority of XF/SF studies, a shorter population doubling time with stable morphology and immunophenotype was reported, compared to traditional FBS cultures. In addition, floating sphere culture [25], a microcarrier-based bioreactor culture system [67], and successful cryopreservation [15, 68] of ASCs in XF/SF conditions have all been reported, supporting the potential applicability of XF/SF culturing conditions.

In conclusion, ASCs in XF/SF culture media show higher proliferation rates compared to those in traditional serum-containing medium, which is essential for clinical cell expansion protocols. However, the proliferation capacity of ASCs may diminish more rapidly in XF/SF conditions compared to serum-containing medium [15]. Thus, population doubling studies at high passages would be justified to investigate the potential early senescence of ASCs in XF/SF media. Although XF/SF media contain patent-protected cocktails of growth factors, additional coating of cell culture plastic is typically used in XF/SF culture [66, 69]. Consequently, cell attachment and differentiation under XF/SF cultures may be insufficient without additional growth factors or coatings. Moreover, the chosen culture condition may direct cell differentiation down a desired lineage [20], and thus, the choice of a culture condition may also depend on the downstream application of the cells [70]. Serum-free alternatives are attractive both scientifically and clinically, but the bulk of experimental data relates to studies performed in serum-based cultures, which—without further investigation—hinders the safety assessment of XF/SF media for regulatory authorities. Thus, XF/SF cultures are currently mainly used for research purposes.

The development of defined XF and/or SF culture protocols is still important for clinical translation of ASCs. Commercially available SF media have been introduced for MSC expansion, of which perhaps the most used media are the STEMPRO® MSC SFM [15, 57, 71], from Life Technologies, and MesenCult™-XF medium [24, 72], from Stem Cell™ Technologies. XF and/or SF culture media are typically offered together with a coating supplement to support XF/SF cell attachment. However, it is important to point out that companies often protect the XF/SF media composition by intellectual property rights, and thus, the detailed composition remains unknown to researchers. Therefore, if manufacture of a certain medium ceases, researchers may have to repeat several steps of product development in order to ensure that the product still has the same properties. This can be a time-consuming and costly process, bringing additional challenges for clinical translation of ASCs.

## 4. Preclinical Characterisation and Allogeneic Use of ASCs

This chapter discusses the strengths and weaknesses of using allogenic ASCs in clinical therapies and will demonstrate the potential of ASCs for immunomodulatory therapies. Moreover, the importance of using appropriate *in vivo* models and bridging the gap from cell culture to clinic is discussed.

### 4.1. Allogeneic Use of ASCs

For practical purposes, cells should be available as an off-the-shelf product immediately upon demand at the point of care [73]. For example, for the treatment of acute ischemic stroke, ASC administration should be performed within the first two weeks of stroke [74]. Generating a therapeutically effective cell dose requires an extended cell expansion phase that is not suitable for the treatment of acute conditions [75]. Moreover, different cell donors have significant variations in the composition of their secretomes and the immunomodulatory capacity of their cells, which may lead to highly variable clinical outcomes.

By using allogeneic ASCs, several or even hundreds of patients could be treated using only one or several cell donors, and optimal cell characteristics could be selected for specific applications. Thus, the use of allogeneic ASCs may be more suitable for clinical demands and represents a step towards commercialisation. Furthermore, the possibility of pooling several donors is an advantage with allogeneic cells. In order to avoid donor-to-donor heterogeneity, pooled MSCs of eight allogeneic donors were used to treat acute graft-versus-host disease, with excellent clinical outcomes [76]. It was demonstrated that a significantly stronger suppressive capacity can be exerted using pooled bone marrow MSCs (BM-MSCs) than using MSCs from the same donors individually [76].

Adipose stem cells are suitable candidates for allogeneic cell therapies due to their low immunogenic profile, which is demonstrated by low expression of major histocompatibility complex (MHC) class II molecules, and T and B cell costimulatory molecules CD80, CD86, and CD40 *in vitro* [3, 4]. When ASCs are used as stimulator cells in a one-way mixed lymphocyte reaction (MLR) assay, ASCs do not stimulate a proliferative response in allogeneic T cells *in vitro* [3, 4, 8, 77]. McIntosh and coworkers demonstrated that the immunogenicity of ASCs decreases with cell passaging, and SVF may remain more immunogenic compared to cells at higher passages [3]. This is probably owing to a more homogenous cell population after immune cells are removed through passaging.

### 4.2. Critical Aspects Related to Allogeneic Use of ASCs

Although ASCs have low immunogenicity *in vitro*, it has been reported that they do elicit a humoral and cellular immune response *in vivo* and thus should not be considered to be fully immune privileged [75]. It is critical to understand that expanded MSCs may not express MHC II *in vitro*, but the expression is likely activated *in vivo* at sites of inflammation [78–80]. Moreover, MSCs express Toll-like receptors (TLR) 1-6 [79], of which TLRs 2-4 are upregulated under inflammatory conditions. TLR activation in MSCs may affect their function and modify their efficacy and survival *in vivo*. Thus, the severity of rejection of allogeneic ASCs is strongly dependent on context and dictated by a balance between cells' expressions of immunogenic and immunosuppressive factors [75]. If ASCs express more immunogenic factors, they may function much like antigen-presenting cells and be able to promote inflammation *in vivo* [75, 81]. In *in vitro* assays, such as MLR, the immunosuppressive properties of ASCs dominate, because the concentration of ASCs is high enough to strongly influence the microenvironment within the cell culture.

Furthermore, ASC differentiation may change the immunogenic profile of the cells, and expression of HLA I and HLA II may significantly increase upon differentiation [82, 83]. Niemeyer and coworkers have reported that undifferentiated ASCs *in vivo* may be excellent candidates for allogeneic cell therapies but that osteogenic-induced cells might be eliminated by the host's immune system [84]. However, contradictory results have also been published, showing that osteogenic induction or osteoinductive biomaterials do not modify the low HLA expression of ASCs [4, 85]. Additionally, we have demonstrated, using an MLR assay, that the culturing condition applied may have an effect on the immunogenic properties of ASCs [86]. In this study, ASCs cultured in FBS medium had the lowest immunogenicity compared with ASCs expanded in HS and XF/SF conditions, but differences were minor. It could be speculated that cells cultured in FBS medium are unable to trigger full immune responses because of the origin of bovine serum, which is harvested from the blood of bovine foetuses with immature immune systems [87].

When autologous and allogenic MSCs derived from BM-MSCs were compared in a clinical trial for their efficacy to treat ischemic cardiomyopathy, an improved efficacy was observed using autologous cells, although no significant donor-specific immune reactions were observed [83]. By contrast, a clinical trial on osteoarthritis and degenerative disc disease has shown that donor-recipient HLA matching of MSCs does not enhance the efficacy of the treatment [82]. Thus, it could be speculated that allogenic MSCs seem to stimulate innate immune responses to some extent, and a certain degree of HLA II matching could be appropriate when using allogeneic ASCs.

The possibility for anti-HLA immunisation is especially critical if subsequent organ transplantation is required. The strengths and weaknesses of allogeneic ASC-based treatments should be critically evaluated, case by case. It could be speculated that allogeneic ASC-based therapy should primarily be used for complicated, time-sensitive and life-threatening conditions such as stroke, whereas non-critical conditions may be treated using autologous cells.

In addition to risks related to HLA immunisation, all stromal cells—including ASCs—are known to express tissue factors on their surface, which may activate the coagulation cascade *in vivo*, and elicit an instant blood-mediated inflammatory reaction (IBMIR) and thromboembolic events after systemic infusion [88]. This may compromise the survival and function of systemically infused ASCs. *Ex vivo* expanded MSCs trigger the IBMIR, both *in vitro* and *in vivo*, and the reaction is dose-dependent and increases with prolonged expansion [88, 89]. It was noticed that a higher cell number also significantly increased clot formation, partially dependent on coagulation factor VII [88]. Nevertheless, the low doses of low-passage MSCs that are typically used in cell therapies elicit only minor systemic effects, but higher cell doses and higher passage cells should be handled with care [89]. The hemocompatibility of ASCs should be carefully examined for patient safety.

In conclusion, allogeneic ASC-based therapy faces significant challenges, but autologous ASC-based therapy is not without problems [75]. The choice between allogeneic versus autologous cells should always be made case by case, considering all potential risks and benefits for each individual patient.

### 4.3. Potential of ASCs for Immunomodulatory Therapies

The immunosuppressive capacities of ASCs are now well recognised within the scientific community [3, 5–9, 86]. In order to achieve effective cell therapies with focused therapeutic effects, it is important to understand the immunosuppressive mechanisms of ASCs at molecular and intercellular levels. Suppression is primarily mediated through a paracrine effect, by modulating the cytokine milieu and lymphocyte functions, e.g., activating regulatory T cells [5, 9, 90]. Immunomodulatory effects may be mediated through macrophage polarisation from proinflammatory M1 phenotype into anti-inflammatory M2 phenotype [91–93]. MSC-mediated immunosuppression includes both soluble factors and direct cell-cell contacts, and additionally, the local cellular environment influences the immune plasticity of MSCs [94–97]. In response to changes in a local cellular environment, immunomodulatory cells, such as regulatory T cells, are activated by anti-inflammatory molecules produced by ASCs [98, 99].

A potential limitation of ASC therapy is that ASCs do not persist following infusion, as the majority of cells have been reported to die within 48 h of systemic infusion [75]. It is still hypothesised that ASCs produce factors that modify the tissue microenvironment, eventually leading to intrinsic recovery, although cells may disappear [99]. Thus, the observed therapeutic effect of ASCs may be due to a hit-and-run mechanism mediated by the production of exosomes or trophic and immunomodulatory factors during the initial days following ASC injection [75, 100]. The therapeutic benefit of ASCs may be reached partly through reprogramming of the immune system using apoptotic cells [75]. Extending the persistence of ASCs after injection, by using immunosuppressive drugs or directly modifying their immunogenicity, is a potential approach to improving their therapeutic effect [75].

In conclusion, ASCs cannot be considered truly immune privileged; rather, there is a balance between expressions of immunogenic and immunosuppressive factors [75]. It has been suggested that—similar to macrophages—MSCs can be polarised into more pro- (MSC1) or anti-inflammatory (MSC2) directions [101, 102]. The final determination of immunomodulatory responses is likely elicited through a combined action of direct cell-cell contacts and secretion of soluble factors, following modulation of the local inflammatory environment. Signalling proteins may play distinct roles, depending on the specific cellular microenvironment. Some of the key functions during MSC-mediated immunomodulation of important signalling proteins is listed in Table 1. The immunosuppressive capacity of ASCs has been discussed in more detail in previous publications [103–105].

### 4.4. Not Lost in Translation: *In Vivo* Studies for Allogeneic ASC-Based Therapies

ASC characteristics observed on cell culture plastic may not fully correlate with those observed in the patient. Clinical phase study attrition rates have remained high, the majority of failures being due to lack of efficacy (56%) or due to safety issues (28%) [143]. Thus, optimisation of *in vivo* models is critical for the successful development of cellular therapies [144]. However, it is challenging to evaluate the functionality of allogenic human ASCs using *in vivo* models, due to differences between human and animal species [145]. For example, human ASCs have a great potential for treating inflammatory diseases, but it has been shown that genomic responses in mouse models poorly mimic human inflammatory diseases [145]. Human ASCs that are transplanted into an animal are not only allogenic but also of xenogeneic origin. To overcome this problem, allogeneic cells isolated from the same species could be utilised but may not give a reliable result of human allogeneic cell function. This is an inherent issue faced in all *in vivo* studies utilising human cells. Nowadays, there are various tissue models available for ex vivo testing, as well as body-on-chip approaches to overcome this problem [146]. Although these models are welcome alternatives, they still are at an early developmental stage and cannot replace animal studies completely, because systemic effects must be studied *in vivo.* In conclusion, the definite functionality of human allogeneic ASCs could only be tested in controlled clinical trials.

Several published animal studies have shown evidence of the safety and efficacy of human ASCs, as recently reviewed [32]. Human ASCs have been successfully tested *in vivo* for the treatment of acute myocardial infarction [147–149], pulmonary diseases [150, 151], and enhanced recovery after stroke [152–154]. Human ASCs are known to secrete several angiogenic [155] and neurogenic factors [156] and to promote vascular maturation [157]. Promising results have also been achieved in various *in vivo* stroke models using different delivery routes [158, 159]. Moreover, the safety and efficacy of human ASCs have been demonstrated in animal models for the treatment of multiple sclerosis [160, 161], glioblastoma [162, 163], spinal fusion [164], chronic liver failure [165, 166], and acute kidney injuries [167, 168]. Furthermore, human ASCs have been successfully used in bone regeneration [73, 169, 170] and for the treatment of acute anal sphincter injuries [171]. The capacity of ASCs to cure inflammatory bowel diseases has been proven, both *in vivo* [10] and in phase III clinical trials. However, safety and efficacy studies should still be performed in controlled clinical trials, in order to ensure the clinical potential of ASCs for the above-mentioned conditions.

Optimisation of *in vivo* models is critical in order to bridge the gap between *in vitro* research and clinical applications [144], but limitations are associated with many of the models used and the challenges of finding an appropriate *in vivo* model that could be reliably translated to human subjects. Moreover, different disease applications require different *in vivo* models. Interpretation of the results may be challenging due to differences between human and animal species [145, 172, 173]. For instance, transcriptional response in mouse models poorly reflects human diseases, due to evolutionary differences between the species, the complexity of the human disease, and the inbred nature of mouse models [145]. In addition, differences in cellular composition between mouse and human tissues may contribute to variation between molecular responses. Furthermore, a different temporal recovery from diseases between patients and mouse models complicates the interpretation of data.

Thus, multiple factors must be considered when designing *in vivo* studies and interpreting data, including the following: (1) the species and strain used (such as mouse, rat, dog, or pig); (2) the status of the immune system (immunocompetent versus immunocompromised); (3) the immunological characteristics of the donor cells (autologous/syngeneic, allogeneic, and xenogeneic); (4) the method, site (intramuscular injection, subcutaneous transplantation), and timing of cell delivery; and (5) the imaging and quantitative methods applied (MRI, nuclear imaging, and histology) [174]. The aim is to provide safe, effective, and reproducible treatments to the patient. Well-designed and standardised clinical trials are necessary to verify the safety and efficacy of ASCs for allogeneic stem cell treatments and immune modulating therapies. Clinical trials for ASCs are described below in more detail.

## 5. Characterisation and Validation of ASCs for Clinical Translation

Adipose stem cell research takes place in a dynamic, rapidly evolving field that requires further standardisation. Thus, guidance in support of safety and biologic clarifications for clinical practices is provided by the International Federation of Adipose Therapeutics (IFATS) and International Society for Cellular Therapy (ISCT) [14]. This chapter introduces the guidelines for immunophenotypic characterisation using cell surface markers, discusses the safety aspects of ASC therapies, and describes the relevance of robust potency assays.

### 5.1. Surface Marker Expression of ASCs

Due to the heterogeneous nature of ASCs, cells should be characterised each time they are used in clinical applications. Phenotypic validation is part of the safety evaluation that ensures that the cell population gained through isolation and expansion steps still expresses the characteristic MSC phenotype. The immunophenotypic analysis should be performed after cell isolation and then be repeated after the expansion phase. The results are used as a criterion for releasing cells for clinical use.

No single markers are available for the recognition of ASCs, but instead, the use of a multicolour identification panel of several cell surface markers is recommended. Additionally, a viability marker is also suggested to eliminate dead or apoptotic cells induced by the isolation procedures. According to recommendations by the IFATS and ISCT, ASCs should be negative (<2%) for hematopoietic markers such as CD14 or CD11b, CD45, CD86, and HLA-DR and positive (>90%) for stromal markers such as CD13, CD73, CD90, and CD105. To distinguish ASCs from BM-MSCs, the use of two additional markers has been proposed, i.e., CD36 (fatty acid translocase) and CD106 (VCAM-1). In contrast to BM-MSCs, ASCs do not express CD106 but are moderately positive for CD36 [78, 175, 176]. Moreover, ASCs have moderate expression of CD34, but the level is greatly dependent on the *in vitro* culture period [177]. It is generally expressed during the early phase of culture, but its expression decreases with continued cell division [176, 177]. Multiple classes of CD34 antibodies exist that recognise unique immunogens, and the choice of CD34 antibody can substantially influence the signal intensity detected on a given cell population. Moreover, the histological analysis of adipose tissue has revealed that CD34-positive cells are primarily associated with vascular structures [178]. Although small numbers of these cells are probably CD31-positive capillary endothelial cells, a CD34+/CD31- cell population of pericytic origin may be derived from adipose tissue [144].

Furthermore, additional markers can further strengthen the characterisation. Bourin and coworkers have suggested that CD10, CD26 (DPPIV), CD49d (VLA4), CD49e (VLA5), and CD146 (MCAM) can be included as additional positive markers, but with variable expression, depending on donor or culture passage. In contrast, low expression (<2%) levels of additional negative markers—CD3, CD11b (Mac-1), CD49f (VLA6), and podocalyxin-like protein—can be observed. Nevertheless, when ASCs are identified using basic surface antigens, it is likely that ASC populations will display heterogeneity for additional surface antigens [78]. Guidelines for immunophenotypic characterisation of ASCs and SVF are summarised in Table 2.

Of note, these characterisation criteria were originally determined for ASCs cultured in traditional FBS culture medium, but the IFATS and ISCT do not take a stand on the effect of serum conditions on cell surface marker expression. Overall, ASC phenotypes seem to be highly similar between cells cultured in standard FBS- or HS-based media versus XF/SF conditions [15, 24]. Mesenchymal stromal markers (CD13, CD73, CD90, and CD105) are strongly expressed in both XF/SF and serum-based conditions, but some minor variations—either increases or decreases, depending on the reference—have been reported with regard to the expression of CD34, CD45, and CD54 in XF/SF conditions [15, 24].

Because ASCs are heterogeneous, it could be speculated that selecting a cell population based on cell surface marker expression may be a useful approach for a specific clinical application, e.g., selecting cells based on CXCR4 or VEGF, to enhance homing or angiogenesis, respectively. It has been demonstrated that homing into an ischemic area was significantly improved among CXCR4-overexpressing ASCs [179] and that VEGF-expressing ASCs had enhanced capacity for blood vessel formation [180]. Thus, phenotypic validation could be used as a method to select a suitable cell population for a specific clinical application. Although this is an attractive approach for achieving an improved clinical outcome, the regulatory authorities will consider this kind of phenotypic validation as an extra manipulation of a cell product, which will hinder safety assessment.

### 5.2. Safety Aspects of ASC Therapies

ASC-based therapies have shown potential for the repair, replacement, or regeneration of damaged cells and tissues. However, a major challenge in cell therapies is ensuring efficacy and safety. During clinical therapies, cells are often expanded *in vitro* outside their natural environment, which may increase the risk for genomic instability or altered differentiation potential. Moreover, there may be an increased risk of significant adverse effects, e.g., tumours and cell growth in ectopic tissues, or severe immune reactions. Genomic characterisation is part of the safety evaluation for ensuring that a cell population that is obtained through isolation and expansion is not contaminated with other cell types and still has a stable genome. Chromosomal tests, including DNA fingerprinting and genomic integrity tests, should be performed after cell isolation, then repeated after the *in vitro* expansion phase, and the results should be used as a release criterion for clinical use.

Cancer treatments generally rely on tumour destruction techniques that may lead to major functional defects in surrounding tissues [181]. This posttherapy damage requires the development of safe regenerative therapies. For breast cancer patients, an autologous fat graft comprising SVF cells is often used as a filler for breast reconstruction to correct possible irregularities after mastectomy [182]. In addition to formal breast reconstruction, ASCs and BM-MSCs favour tissue-healing processes and promote local tissue repair by modulation of the tissue microenvironment [183]. However, interactions between MSCs and cancer cells in modulation of the tumour microenvironment are critical for safety matters. Many components that are required for successful regenerative therapy, such as revascularisation, immunosuppression, and cellular homing, are also critical for tumour progression and metastasis [184, 185]. MSCs are known to secrete cytokines, chemokines, and growth factors that are essential for the development and maintenance of an inflammatory state, thus inducing tissue regeneration after injury [182, 183, 186]. However, these inflammatory responses and paracrine signals stimulated by MSCs may create an optimal microenvironment for cancer cells, which may be induced for continuous proliferation and tumour neoangiogenesis [185, 187–189]. However, it should be highlighted that ASCs do not trigger malignant transformation or initiate cancer. Consequently, ASCs are not inherently tumourigenic, but they may provoke a tumourigenic potential in the presence of certain c-Met-expressing breast cancer cells [182]. This model was presented by Eterno and coworkers, suggesting that c-Met could be used as a marker to predict the risk of cancer recurrence when applying ASCs in cancer patients for regenerative and reconstructive purposes.

In conclusion, the effects of MSCs on tumour cells are multiple and may depend on the state of the tumour cell, the properties of the MSC populations used, and interactions with other cell types, such as tumour-infiltrating immune cells [185]. Several published clinical studies have shown that ASCs do not increase the risk of cancer initiation or progression compared with the control group [190–192], but additional studies are still needed to clarify the crosstalk between aggressive cancer cells and MSCs. A registry of patients receiving ASC treatments would be helpful to monitor long-term outcomes in the context of cancer.

Another important safety concern in the clinical translation of ASCs is the possibility for genomic instability of ex vivo expanded ASCs, i.e., whether they may undergo spontaneous transformation *in vitro*. However, cultured ASCs are reported to be genomically stable in long-term cultures after multiple cell doublings, thus supporting their suitability for regenerative applications [193–196]. Moreover, it has been reported that G-banding analysis may be unsuitable for the detection of low frequency chromosome number alterations, and to increase the rigor of the analysis, fluorescence in situ hybridisation (FISH) analysis should be performed for effective detection. The influence of clinical grade human PL on the genomic stability of ASCs has also been investigated [193], showing that ASCs preserve their normal genotype when cultured under XF condition. In long-term (6 months) genomic stability tests, some minor deletions in gene-rich telomeric regions have been observed in the early passage in the ASC subpopulation, but they were spontaneously eliminated and cells remained genomically stable [197].

Around ten years ago, it was reported that human MSCs undergo spontaneous transformation into cancerous cells [198, 199]. These studies were later withdrawn, as it was shown that the cells used in the transformation studies were cross-contaminated by cancerous cells that initially grew slowly in the presence of human MSCs [200]. It was proved by DNA fingerprinting and short tandem repeat analysis that the transformed MSCs had been cross-contaminated by human fibrosarcoma, osteosarcoma, or glioma cell lines [200, 201]. These observations highlighted the need for extremely rigorous cell culture procedures when utilising primary cell cultures for therapeutic purposes. Moreover, clinical safety and efficacy studies should be performed before further clinical use, in order to avoid any adverse effects connected with cell-based therapies.

### 5.3. Potency Assays for Evaluating ASC Functionality

A final cell product—particularly its active substances—must be characterised to a sufficient level that ensures that only a safe and efficient product will be administered to a patient [202]. In addition to measuring safety and efficacy, a potency assay is used as a tool to test the cell product's stability and variation between batches. These functional tests should be performed during *in vitro* expansion before a cell product is released for clinical use.

Identification of relevant and robust potency assays is not only a regulatory requirement, but they provide a solid basis for producing and delivering a product that is consistent, safe, and ultimately therapeutically effective [203]. Potency can be defined as the ability of a treatment to elicit a particular response at a certain dose, and thus, it is a quantitative measure of a relevant biologic function based on attributes linked to relevant biologic properties. Although ASCs derived from different donors would have similar morphologic, immunophenotypic, and differentiation characteristics, they may still have major differences in their biologic and functional attributes. The ISCT has recently identified three preferred analytic methods that could be utilised as a matrix assay approach: (1) quantitative RNA analysis of selected gene products, (2) flow cytometry of functionally relevant surface markers, and (3) protein-based assay of secretome [204].

According to the ISCT, there is no single test that can adequately measure product attributes that predict clinical efficacy. Considering this limitation, the potency assay should measure the product's mechanism of action, i.e., relevant therapeutic activity or intended biological effect. However, there are challenges connected with this approach. The MOA of a cell product may be complex and incompletely characterised, or it may have multiple active ingredients and biological activities that are difficult to specify at an early phase of a clinical study. For example, an MOA may partly rely on differentiation capacity, but simultaneously, paracrine factors may have a role. A cell product may also have plasticity or limited stability that will complicate the development of a robust potency assay. For these reasons, the above-mentioned analytic methods, referred to as the matrix assay approach, would be recommended and more straightforward to perform.

In order to evaluate the MOA of ASCs for immunomodulatory therapies, the use of functional *in vitro* assays with responder immune cells would be one option. The MOA could be evaluated using allogeneic human peripheral blood mononuclear cells. However, this approach also has limitations. Although activated T cells provide an opportunity to measure proliferation inhibition and cytokine production *in vitro*, it is not known whether this assay accurately reflects the MOA of ASCs *in vivo* [204]. ASCs affect the cell physiology of monocytes, B cells, natural killer cells, and granulocytes, which are not studied in a classic MLR assay performed with solely T cells as responders. Moreover, relative to their homeostatic resting state, ASCs undergo polarisation toward immunosuppressive phenotype on exposure to various proinflammatory cytokines, such as interferon- (IFN-) *γ*, tumour necrosis factor *α*, IL-1*α*, or IL-1*β* [204]. This immune plasticity is visible using *in vitro* licensing that better recapitulates what likely happens *in vivo* when ASCs are transfused into patients. Thus, comparing results with both resting and licensed ASCs would be the most informative when aiming for clinical use [204]. For example, licensing with IFN-*γ* for 12–48 h is adequate to obtain cell activation that allows for their analysis as part of an assay matrix.

Overall, appropriate potency assays for MSCs are essential tools for verifying the comparability of MSC products, but their development remains challenging. Quantitative data on a cellular product and how it exerts specific effects at a certain dosage are important information in the progress of developing it into a cell-based therapy [203].

## 6. Clinical Studies for Evaluation of ASC Potential

**Figure 2:**
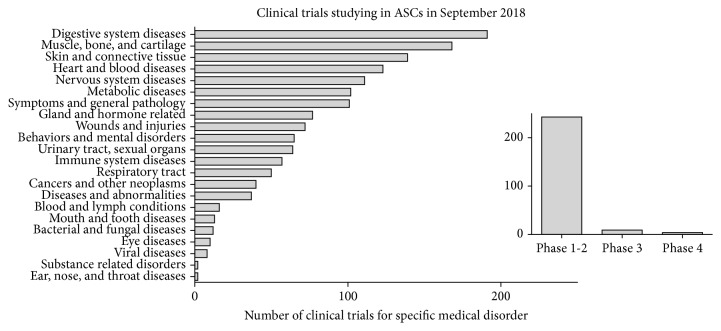
A total number of 282 clinical trials using ASC were ongoing on the 11th of September 2018, based on http://www.clinicaltrials.gov. Clinical studies were categorized based on the disease or target tissue of the treatment. Only 13 out of 282 trials had progressed into phase III or IV. A commercial sponsor was involved in 116 trials.

In September 2018, a total of 282 clinical trials evaluating the potential of ASCs for treating different types of medical disorders were found at http://www.clinicaltrials.gov (Figure 2). However, all of them did not use expanded ASCs; SVF was used in at least 22 trials (8%). Some of these 22 trials also used the term ASCs, and thus, the terminology used may be misleading in some cases. Only 13 trials (5%) progressed to phase III or IV (Figure 2). A commercial sponsor was involved in 116 trials (41%), whereas the remainder of trials were conducted in academic or hospital settings. The two most common cell therapy applications of ASCs were the treatment of joint disorders, such as osteoarthritis, as well as treatments of gastrointestinal diseases, especially complex fistulas that are often associated with Crohn's disease. Considering the chronic inflammatory status that is connected with the above-mentioned diseases, the MOA of ASC therapies seems to be immunomodulatory and paracrine effects. Furthermore, ASCs have been widely applied in clinical trials concerning skin and connective tissue diseases, heart and blood diseases, nervous system diseases, and nutritional and metabolic diseases. Many of the trials focussed on multiple disease applications.

### 6.1. Good Manufacturing Practice Regulations

GMP facilities that perform advanced cell manipulation must be well controlled, in order to ensure safe and efficient cell therapies for patients. In Europe, the Committee for Advanced Therapies (CAT) at the EMA regulates the use of ASC-based tissue engineering products that are defined as advanced therapy medicinal products (ATMPs) [205]. ATMPs are medicines for human use based on gene therapy, somatic-cell therapy, or tissue engineering [206] and are often at the forefront of innovation, offering potential treatment opportunities for diseases that currently have limited or no effective therapeutic options [205]. Regulations on ATMPs provide a consistent legal system covering the collection, testing, processing, storage, and distribution of human tissues, cells, and blood, as well as the manufacturing of ATMPs made from human materials. EMA regulations are similar to the regulatory framework set up by the Food and Drug Administration (FDA) in the United States [207].

Both FDA and EMA implement a risk-based approach to regulation and classify procedures according to the degree of manipulation involved and the risk of adverse processing-related events [205, 207]. A risk-based approach focuses on three general issues: limiting the risk of transmission of disease from donors to recipients, establishing manufacturing practices that minimise the risk of contamination, and requiring an appropriate demonstration of safety and effectiveness. Minimal manipulation can be performed using good tissue practices (GTPs), which is a less-defined standard used mostly in industry. Processes classified as substantial manipulation require a higher degree of process control, designated as GMPs [205, 208, 209]. The term “substantial manipulation” denotes that the biological characteristics, functions, or properties relevant for the therapeutic effect have been altered [208]. This includes, for example, ex vivo expansion, activation or combination with nontissue components, or use in an application other than that of the tissue's normal function. The current regulatory landscape considers ASC isolation from adipose tissue alone to be substantial manipulation, hindering the use of freshly isolated SVFs without GMP.

## 7. ASC-Based Products with Marketing Authorisation

Although clinical trials are a required step forward in clinical translation, licensed products and those approaching marketing authorisation are still few in number. Only seven ATMPs—classified as cell therapy medicinal products or tissue-engineered products—have received marketing authorisation in Europe (Table 3). Only four of these products currently hold marketing authorisation, since developers of three products have withdrawn their authorisation due to commercial reasons. ATMPs often target orphan diseases, because the development of orphan products is financially well supported by EMA, but consequently, these products have relatively small markets. Therefore, it may be difficult to make a profit on ATMPs, making the incentive to continue producing them limited. Before receiving marketing authorisation, a major challenge in commercialisation is the manufacturing and quality assurance of cell-based products [202]. Demonstration of quality, safety, and efficacy of cell-based products is extremely costly and demanding, because it is difficult to ensure comparability between production processes and batches [202]. In addition to safety and efficacy demonstration, cell-based products should be cost-effective, in order for them to be accessible to patients and for their provision by public healthcare services to be feasible [210].

After a decade-long development, the first ASC-based product received a marketing authorisation in the EU in March 2018 (http://www.ema.europa.eu). Alofisel®, produced by cell therapy company TiGenix, is a medicine that is used to treat complex anal fistulas in adults with Crohn's disease. This cell therapy product contains 120 million expanded allogeneic ASCs that are applied locally as a single injection in perianal fistula tracts [34, 211]. In a phase III clinical trial, after one-year follow-up, the product was found to be efficacious at inducing and maintaining fistula closure, compared to placebo (http://www.clinicaltrials.gov, NCT01541579). Alofisel®'s MOA has not been elucidated in human studies, but in preclinical studies, it was shown to have immunomodulatory and anti-inflammatory effects at inflammation sites. These effects are mediated through induction of indoleamine 2,3-dioxygenase, resulting in impaired proliferation of activated lymphocytes, and reduction of inflammatory cytokines [212].

**Figure 3:**
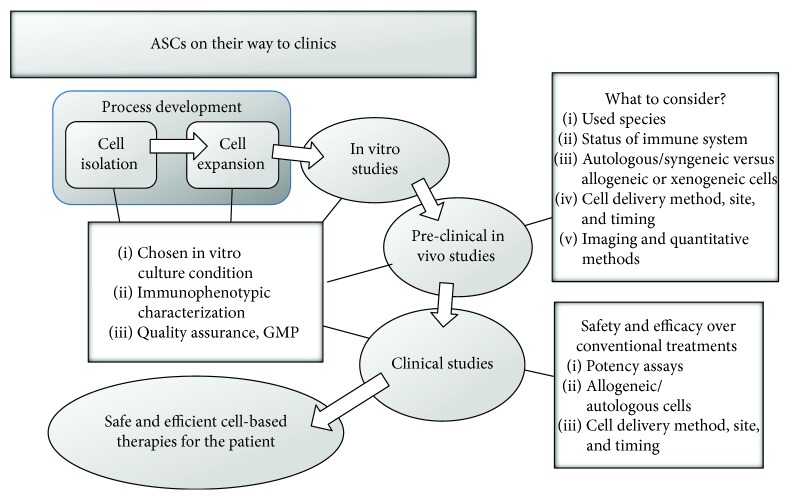
Required steps during clinical translation of ASCs.

In addition to Alofisel®, TiGenix is focusing on developing and commercialising other ASC-based therapeutics (http://www.tigenix.com). The company has a clinical stage pipeline consisting of two ASC programmes in which the applicability of allogeneic ASCs is being investigated in clinical trials for the treatment of severe sepsis (NCT02328612; http://www.clinicaltrials.gov) and autoimmune diseases via intralymphatic administration (NCT01743222; http://www.clinicaltrials.gov). ASCs offer promise for future medical applications, but only time and further research will tell the final outcome of these complex medicinal products.  Figure 3 summarises the different stages required for clinical translation of ASCs.

## 8. The Costs of Clinical Cell Therapies

The demonstration of safety and efficacy of ASC-based therapy is a costly process for the investigator; however, the costs of cell-based therapies are high also for public health systems worldwide. The future of cell-based therapies, including financial issues, was recently discussed at the Lancet Commission [213]. Many potential cell-based therapies will have substantial costs when delivered to patients. These costs will include not only hospital costs (surgeries, postoperative treatment, and follow-up) but also the costs of cell expansion in clean room facilities and sterility and safety testing. Compared with conventional treatments, the costs of cell-based therapies may be substantially higher. However, they may be offset by potential savings over the longer term, by reducing the need for expensive health and social care, especially for chronic and life-limiting illnesses such as Crohn's disease [213]. Cell-based therapies could improve the quality of life of many patients with chronic diseases and, additionally, could have the major impact of reducing the demand for healthcare.

The progress of cell-based interventions is based on decisions made by patients, healthcare professionals, and payers. Key factors that influence such decisions include the known risks and benefits of a cell-based treatment option, individual preferences of patients, and treatment providers, as well as availability and cost. It should be recognised that—along with safety, efficacy, and accessibility—the economic value is an important measure of the overall utility of any therapeutic and is often a deciding factor. The cost of treatment should not prevent patients from accessing stem cell-based interventions for life-threatening or seriously debilitating medical conditions. Safe and efficacious therapeutics should be accessible to any patient in need, irrespective of financial status.

## 9. The ASC Patent Landscape

Until recently, stem cell research conducted within academic settings has paid no or little attention to patent questions. However, academic researchers are increasingly focussing on patenting matters as universities become more involved in commercially sponsored research projects and projects heading for clinical applications [214]. In the field of stem cell research, patents may cover, for example, instruments for cell isolation or preparation, optimised culture conditions with defined growth factors, proteins or small molecules, or methods for expansion and differentiation or dedifferentiation of cells. Many of these methods are separately patented technologies that have few alternatives and therefore may be protected by intellectual property rights. Celution® system is an example of a patented technology that has been developed for clinical use to facilitate automated processing of ASCs in a functionally closed system [215].

To provide an overview of the latest inventions in the field, investigation of patent applications is a fast and useful method for gaining access to the latest data. In this review, a patent search was performed using the Espacenet Worldwide database (http://www.epo.org) that covers patents and patent applications from over 90 countries (conducted on the 20th of September 2018). When a patent search was conducted using the keywords “adipose stem cell” in the patent title or abstract, 863 hits were found, whereas the keywords “adipose stromal cell” produced 193 hits. With a more detailed combination of keywords, “adipose stem cell clinical” and “adipose stromal cell clinical” returned 46 and 10 hits, respectively. These patents included protocols for isolation, expansion, cryopreservation, and differentiation of ASCs for clinical use. Patents for reprogramming ASCs into iPSCs were also found, as well as clinical application methods for treating fistulas, skin wounds, myocardial infarction, bone defects, or dental injuries using ASCs. The search results are summarised in Table 4.

The technical content of the patent landscape is highly complex. As the number of patent applications increases in the field, access to scientific knowledge is potentially limited. Open access publishing is highly recommended nowadays, and access to data and materials is critical for the progress of stem cell science and cell therapies. A number of factors currently limit the sharing of data, including ethical regulations concerning the use of human cell lines, the complexity of the landscape of intellectual property rights, and the potential competitive spirit of individual scientists [216]. Although these potential obstacles should be kept in mind when patenting, at the same time, it is justifiable to offer protection to researchers who invest in the development of products and processes that have significant medical benefits.

The original patent that covers ASCs was published in 2004 by Katz and coworkers [217]. This particular patent was broad and covered the cells both alone and within biologically compatible materials, as well as the methods used to generate differentiated tissues and structures, both *in vivo* and *in vitro*. Additionally, Katz et al.'s patent included the secreted hormones and conditioned media of ASC cultures, as well as the production of an extracellular matrix lattice from adipose tissue. However, due to prior art in the academic literature, Katz et al.'s patent claims were challenged and overturned in the US patent court. Because this particular overturned patent is a prior art for all subsequent patents on the use of ASCs, the current patent holders may not be able to withhold licensing of ASC-derived methods or restrain freedom to operate in the future. The above-mentioned detail may change the patent landscape for SVF and ASCs in the coming years.

## 10. Future Perspectives

In conclusion, ASCs represent an alternative treatment for a wide range of human diseases that have limited or no effective therapeutic options. These cells are especially effective for treating inflammatory or autoimmune diseases. ASCs have been shown to home in on the injured tissues, and the therapeutic efficacy is primarily mediated through paracrine effects.

The clinical translation of ASCs is a time-consuming process, and the cells are still not in routine clinical use, although the first ASC-based product has received marketing authorisation in the EU. An increasing number of *in vitro* and *in vivo* reports, as well as clinical trials, study the applicability of ASCs, but some critical issues remain and should be resolved prior to clinical development. As highlighted previously in this review, the use of allogeneic cells would be recommended for two reasons. ASC characteristics are strongly donor-dependent, requiring precharacterisation of the cell population. In addition, acute conditions such as stroke require immediate cell therapy, where time-consuming cell isolation, characterisation, and expansion steps are not an option. Moreover, there is still a need for better methods to characterise and validate the cells, as a CD marker profile may not sufficiently identify these complex entities. The development of adequate potency assays will help in the process, although it may be difficult to identify these MOA-based assays for heterogeneous populations of ASCs.

Both SVF and ASCs show functionality in cell-based therapies, but selecting between these two cell populations should be based on the particular disease application. Attention should be paid also to terminology and distinguishing ASCs, which are relatively homogeneous, from SVF, which contain several cell populations. Moreover, the clinical outcome of different disease applications depends on the appropriate administration methods, dose, and timing. In addition to safety and efficacy demonstration, it is relevant to highlight that cell-based products should be made cost-effective, in order for them to be accessible to patients. The future currently looks bright for ASC-based therapies and patients potentially receiving these novel treatments. Ultimately, however, ongoing and upcoming controlled clinical trials will determine the outcome of ASC-based therapies.

## Figures and Tables

**Table 1:** Signalling proteins and some of their functions in MSC-mediated immunomodulation.

Signalling protein	Abbreviation	Function
Interferon *γ*	IFN-*γ*	Stimulates MSCs to elicit immunosuppressive factors [106]; immunomodulatory functions [85, 107]; immunosuppression [90, 96]; induces adhesion molecule expression [95, 96]; regulates chemokine expression [108]
Tumour necrosis factor *α*	TNF-*α*	Immunomodulatory functions [85, 107]; immunosuppression [90, 96]; regulates chemokine expression [108]
Indoleamine 2,3-deoxygenase	IDO	Immunosuppression [90, 109–111]; inhibits T cell proliferation [112]; promotes type II macrophage differentiation [113]; impairs NK cell activity [114]
Prostaglandin E2	PGE2	Immunosuppression [5, 110, 115, 116]; induces Foxp3+ Tregs [117]; inhibits NK cell function [116, 118]; induces type M2 macrophages [92]; inhibits dendritic cell maturation [119]
Galectin-1	Gal-1	Immunosuppression [120–122]; inhibits T cell proliferation [123]; modulates release of cytokines, such as TNF-*α*, IFN-*γ*, IL-2, and IL-10 [120]
Galectin-3	Gal-3	Immunosuppression [121, 122, 124]; induces T cell proliferation [124]
Transforming growth factor *β*1	TGF-*β*1	Multiple actions in innate and adaptive immunity, important factor in maintaining immune tolerance [125]; immunosuppression, suppresses T cells and several cytokines, such as TNF-*α* and IFN-*γ* [110, 126–128]; induces T regs [129]; inhibits NK cell activation and function [116]
Interleukin 6	IL-6	Supports or suppresses inflammation, depending on context [130]; prevents monocyte differentiation toward antigen-presenting cells [131]; inhibits T cell proliferation [132]; inhibits dendritic cell differentiation [133]; anti-inflammatory effects mediated through inhibition of TNF-*α* [134]
Interleukin 10	IL-10	Inhibits T cell responses, decreases Th17 cell differentiation [135, 136]
Interleukin 8	CXCL8/IL-8	Induces extracellular matrix degradation [137]; promotes angiogenesis [138]; regulates neutrophil and mast cell functions [139]
C-C chemokine ligands 2 and 5	CCL2/MCP-1 CCL5/RANTES	Promote T cell chemotaxis, attract immune cell or MSC migration to sites of injury or inflammation [137]; induce extracellular matrix degradation [137]; regulate monocyte and effector and memory T cell functions [139]; CCL2 regulates monocyte mobilisation and macrophage infiltration [140]; CCL5 has T cell co-stimulatory functions [141]
CXC chemokine ligand 10	CXCL10/IP-10	Induces MSC migration to inflammation sites [108, 142]; regulates dendritic cells and effector, memory, and regulatory T cell functions [139]

**Table 2:** Guidelines for immunophenotypic characterisation of adipose tissue-derived cells, modified from Bourin et al.'s study [14].

Feature	Assay	Cells of SVF	ASCs
Immuno-phenotype	Flow cytometry	Primary stable positive markers for stromal cells: CD13, CD29, CD44, CD73, CD90 (>40%), CD34 (<20%) Primary negative markers for stromal cells: CD31 (<20%), CD45 (<50%)	Primary stable positive markers: CD13, CD29, CD44, CD73, CD90, CD105 (>80% in ASC)
			Primary unstable positive marker: CD34 (present at variable levels)
			Primary negative markers: CD31, CD45, CD235a (<2%)
			Secondary other positive markers: CD10, CD26, CD36, CD49d, CD49e
			Secondary other low or negative markers: CD3, CD11b, CD49f, CD106, PODXL

**Table 3:** Marketing authorisation approved advanced therapy medicinal products, classified as cell therapy medicinal products or tissue-engineered products, in Europe in October 2018.

Product name	Developer	Active substance	Indication	Approval	Status
Alofisel	TiGenix	*Ex vivo* expanded human allogeneic ASCs	Perianal fistulas in Crohn's disease	2018	Approved
Spherox	CO.DON	Spheroids of human autologous matrix-associated chondrocytes	Cartilage defects in the knee	2017	Approved
Zalmoxis	MolMed	Genetically modified human allogeneic T cells	Stem cell transplantation in high-risk blood cancer	2016	Approved
Holoclar	Chiesi	*Ex vivo* expanded autologous corneal epithelial cells containing stem cells	Severe limbal stem cell deficiency in the eye	2015	Approved
Provenge	Dendreon	Autologous peripheral blood mononuclear cells activated with prostatic acid phosphatase granulocyte-macrophage colony-stimulating factor	Metastatic prostate cancer	2013	Withdrawn in 2015
MACI	Vericel	Matrix-applied autologous cultured chondrocytes	Cartilage defects in the knee	2013	Withdrawn in 2014
Chondrocelect	TiGenix	*Ex vivo* expanded autologous cartilage cells expressing specific marker proteins	Cartilage defects	2009	Withdrawn in 2016

**Table 4:** Patents relating to adipose stromal/stem cells.

Keywords in title/abstract	Number of hits	Patent
Adipose stem cell; clinical	15	Isolation and expansion or cryopreservation of ASCs
	5	Differentiation induction media for ASCs
	1	Media for reprogramming ASCs into iPSCs
	11	Clinical application methods for treating: (1) fistulas; (2) skin wounds; (3) atopic dermatitis; (4) soft tissue injuries; (5) erectile dysfunction; (6) dental injuries; (7) hair loss, using ASCs
	3	Conditioned medium for reducing inflammation
	1	Method for propagating serum-derived hepatitis C virus using ASCs
	2	Scaffolds for ASCs: (1) myocyte-mixed sheet scaffold for clinical applications; (2) porous scaffold for bone tissue engineering applications
	1	Culture system for bone tissue engineering
	2	Method for promoting ASC adhesion, migration, homing, and angiogenesis
	3	Extracellular matrix or acellular matrix for clinical applications
	1	Method for constructing tissue-engineered blood vessel
	2	*Ex vivo* model for (1) vascular malformation and (2) myocardium infarction

Adipose stromal cell; clinical	4	Isolation, expansion, or cryopreservation of ASCs for clinical use
	2	Differentiation induction media for ASCs
	4	Clinical application methods for treating: (1) fistulas; (2) skin wounds; (3) dental injuries, using ASCs

Adipose stem cell	871	

Adipose stromal cell	195	
